# Dynasore impairs VEGFR2 signalling in an endocytosis-independent manner

**DOI:** 10.1038/srep45035

**Published:** 2017-03-22

**Authors:** Dimitris Basagiannis, Sofia Zografou, Katerina Galanopoulou, Savvas Christoforidis

**Affiliations:** 1 Institute of Molecular Biology and Biotechnology-Biomedical Research, Foundation for Research and Technology, 45110 Ioannina, Greece; 2 Laboratory of Biological Chemistry, Department of Medicine, School of Health Sciences, University of Ioannina, 45110 Ioannina, Greece

## Abstract

VEGFR2 is a critical angiogenic receptor playing a key role in vascular homeostasis. Upon activation by VEGF, VEGFR2 becomes endocytosed. Internalisation of VEGFR2 is facilitated, in part, through clathrin mediated endocytosis (CME), the role of which in VEGFR2 function is debated. Here, we confirm the contribution of CME in VEGFR2 uptake. However, curiously, we find that different approaches of inhibition of CME exert contradictory effects on VEGF signalling; knockdown of clathrin, or of dynamin, or overexpression of dynamin K44A, do not affect VEGF-induced phosphorylation of ERK1/2, while dynasore causes strong inhibition. We resolve this discrepancy by showing that although dynasore inhibits CME of VEGFR2, its inhibitory action in ERK1/2 phosphorylation is not related to attenuation of VEGFR2 endocytosis; it is rather due to an off-target effect of the drug. Dynasore inhibits VEGF-induced calcium release, a signalling event that lies upstream of ERK1/2, which implies that this effect could be responsible, at least in part, for the inhibitory action of the drug on VEGF-to-ERK1/2 signalling. These results raise caution that although dynasore is specific in inhibiting clathrin- and dynamin-mediated endocytosis, it may also exert off-target effects on signalling molecules, hence influencing the interpretation of the role of endocytosis in signalling.

Although binding of extracellular stimulants to their receptors takes place at the plasma membrane, subsequent internalisation of the ligand/receptor complexes is an essential regulatory mechanism that controls the specificity, amplitude and duration of the signalling events^1^,^2^,^3^,^4^. Despite the fact that the list of distinct endocytic pathways is ever growing, clathrin mediated endocytosis (CME) is the most well-described pathway^5^. Among the numerous molecules that have been found to participate in the generation of clathrin coated vesicles, the large GTPase dynamin has been one of the most extensively studied^5^,^6^,^7^. Dynamin plays critical role in mediating the last step of the generation of clathrin coated vesicles, that is, the pinching of the clathrin coated pits^5^,^8^. Given the importance of dynamin in endocytosis, recent studies have generated novel tools (small molecule inhibitors) against this GTPase^9^,^10^,^11^,^12^. These tools have been used extensively in studying the role of clathrin- and dynamin- mediated endocytosis in diverse cellular functions^13^. Among these drugs, dynasore^9^ has been the most widely used^13^.

VEGFR2 is a member of the family of receptor tyrosine kinases that is expressed dominantly in vascular endothelial cells. It is one of the most potent pro-angiogenic receptors and a key molecular player in the pathophysiology of the vascular system^14^,^15^. Given the pivotal role of VEGFR2 signalling in vascular homeostasis, as well as in cancer progression and other angiogenesis-related diseases, unraveling the underlying mechanisms that govern VEGFR2 endocytosis has been imperative for the comprehension of vascular pathogenesis and for targeted therapy^16^,^17^. Although the main VEGF-induced endocytic route of VEGR2 is macropinocytosis^18^, which plays critical role in VEGF functions^18^, a part of the receptor is also internalised via clathrin- and dynamin-dependent endocytosis^18^,^19^,^20^,^21^,^22^,^23^,^24^,^25^,^26^,^27^,^28^,^29^,^30^,^31^,^32^. Intriguingly, the role of this route in the regulation of VEGFR2 signalling remains controversial. Thus, on one hand, knockdown of clathrin or of other molecules of the clathrin machinery have no effect or they augment VEGF-induced activation of ERK1/2^18^,^21^,^22^,^24^,^28^,^29^, yet, on the other hand, dynasore attenuates VEGFR2 signalling^23^,^26^,^33^,^34^. Intriguingly, given that small molecule inhibitors may have off-target effects^13^,^35^, it is unclear whether the inhibitory effect of dynasore in VEGF signalling is due to interference with endocytosis itself or due to concomitant modulation of other molecules that participate in the signalling process (i.e. off-target effects).

To shed light on the above contradictions, we revisited here the role of clathrin- and dynamin-dependent endocytosis on VEGFR2 signalling, using knockdown, protein overexpression, and drug-based approaches, in primary human umbilical vein endothelial cells. Our data show that clathrin or dynamin knockdown, or overexpression of dynamin K44A, do not interfere with VEGF-induced activation of ERK1/2. However, treatment with dynasore, which has been commonly used to interfere with CME of VEGFR2^23^,^26^,^33^,^34^, causes a strong inhibitory effect. To clarify whether the effect of dynasore is due to interference with endocytosis itself, or due to an off-target effect of the drug, we developed a protocol that uncouples the endocytosis-dependent effect of the drug from its possible off-target effects. Our data suggest that although dynasore does inhibit clathrin- and dynamin-dependent endocytosis of VEGFR2, its effect on VEGF-to-ERK1/2 signalling is independent of receptor endocytosis or dynamin; it is rather due to an off-target effect of the drug in signalling. Dynasore inhibits VEGF-stimulated calcium release, an upstream event of ERK1/2 activation, suggesting that the inhibitory effect of dynasore on ERK1/2 could be due, at least in part, to an interference of the drug with calcium release. These data imply that previous findings that were based on the use of dynasore in signalling assays, for a number of different cell surface receptors, should be revisited.

## Results

### Treatment with siRNAs against clathrin, or with dynasore, cause consistent inhibition of CME of VEGFR2

**Figure 1:**
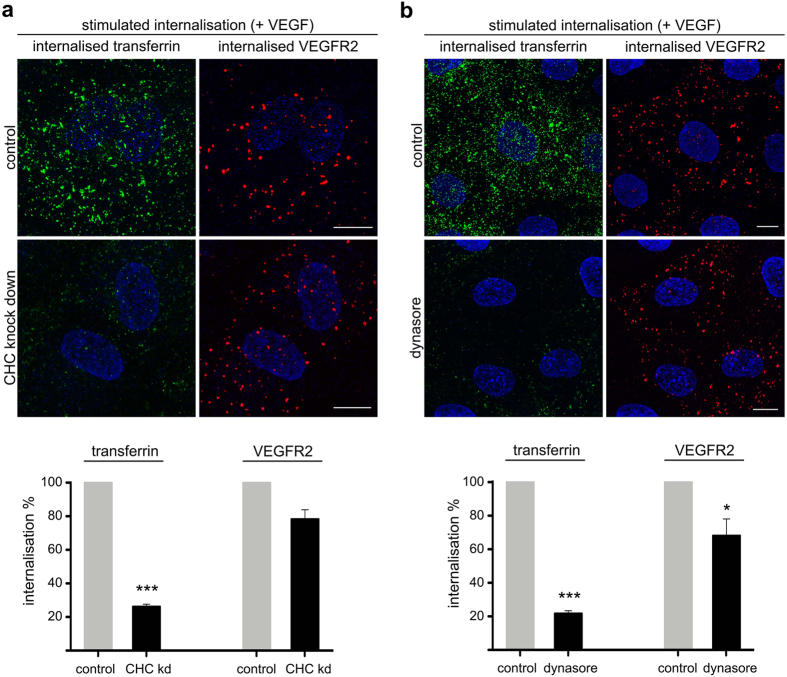
Clathrin-mediated endocytosis of VEGFR2 is consistently inhibited by either knockdown of clathrin or by treatment with dynasore. (**a**) Serum starved (2 h) HUVECs, that were transfected with siRNAs against CHC, were incubated with a mouse anti-VEGFR2 extracellular domain antibody at 4 °C, transferred to 37 °C and stimulated with VEGF, in the presence of FITC-transferrin. Prior to fixation, membrane bound antibodies and transferrin were removed by acid wash and the internalised receptor was revealed by secondary fluorescent antibodies, using confocal microscopy. Nuclei are shown in blue (10 μm scale bars). Quantification of VEGFR2 internalisation and transferrin is shown at the bottom of the immunofluorescence images (20 cells from 3 independent experiments were analysed, mean ± S.D., ***P < 0.001, t-test). (**b**) 2 h serum starved HUVECs were incubated with a mouse anti-VEGFR2 extracellular domain antibody at 4 °C, treated with vehicle (control) or dynasore at 4 °C for 30 min, transferred to 37 °C and processed as described in “a”. Nuclei are shown in blue (10 μm scale bars). Quantification of VEGFR2 internalisation and transferrin is shown at the bottom of the immunofluorescence images (20 cells from 3 independent experiments were analysed, mean ± S.D., *P < 0.05, ***P < 0.001, t-test).

To illuminate previous inconsistencies regarding the role of CME in VEGFR2 signalling, at first we validated the effect of clathrin knockdown, or of dynasore (a widely used dynamin inhibitor^9^,^13^, which has been systematically used to interfere with CME of VEGFR2^23^,^26^,^27^,^33^,^34^,^36^), on VEGFR2 internalisation. To assess the amount of internalised VEGFR2, we followed an anti-VEGFR2 antibody uptake protocol, followed by analysis by confocal microscopy^18^,^21^,^23^,^26^,^37^, in primary human umbilical vein endothelial cells (HUVECs). This method reveals the molecules of newly internalised VEGFR2, while it excludes the non-internalised and intracellular pools of the receptor. Either long-term inhibition of CME by knocking down of CHC (Fig. 1a), or rapid inhibition by treatment with dynasore (Fig. 1b), caused a partial inhibition of VEGF-induced internalisation of VEGFR2 (Fig. 1a,b). Given that both tools (siRNA of CHC and dynasore) inhibited effectively the uptake of transferrin (a typical cargo-marker of CME) (Fig. 1a,b), it is concluded that partial inhibition of VEGFR2 internalisation (by the above tools) is not due to insufficient blockage of CME, but rather due to a partial contribution of this pathway in VEGFR2 internalisation, which is in line with previous reports^18^,^19^,^24^,^26^,^28^. Furthermore, since CHC knockdown and dynasore inhibited internalisation of VEGFR2 to the same extent (up to 20–30%), we conclude that these tools are consistent in blocking CME of VEGFR2.

### Dynasore attenuates VEGF signalling in a clathrin- and dynamin-independent manner

**Figure 2:**
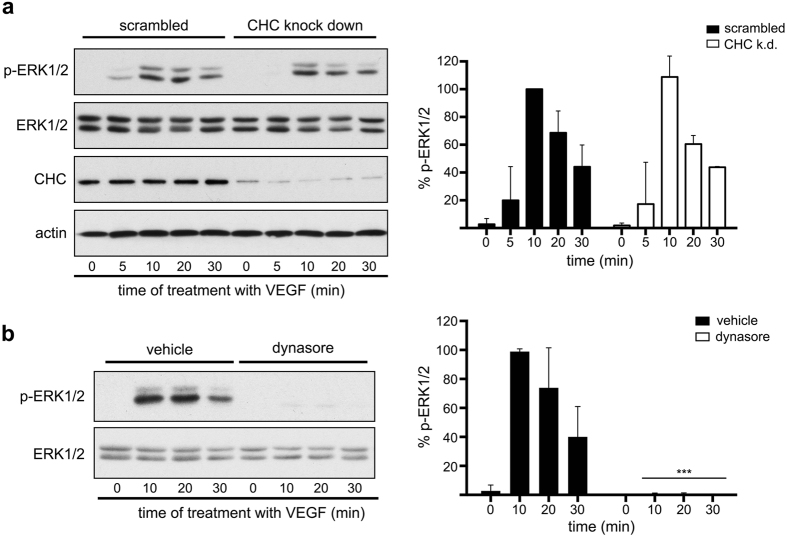
Although knockdown of CHC does not affect VEGF-to-ERK1/2 signalling, dynasore causes a strong inhibitory effect. (**a**) HUVECs transfected with siRNAs against clathrin heavy chain were stimulated with VEGF for various time points, lysed and subjected to immunoblotting analysis using antibodies against the phosphorylated or total ERK1/2. Quantification of the effect of clathrin knockdown on the VEGF-induced phosphorylation of ERK1/2 is shown on the right of the immunoblots. The effect is considered non-significant (n = 3, mean ± S.E.M., ANOVA followed by Dunnett’s analysis). (**b**) Serum starved (2 h) HUVECs were treated with vehicle or dynasore (100 μM), stimulated with VEGF for various time points, lysed and subjected to immunoblotting analysis using antibodies against the phosphorylated or total ERK1/2. Quantification of the effect of dynasore treatment on the VEGF-induced phosphorylation of ERK1/2 is shown on the right of the immunoblots (n = 3, mean ± S.E.M., P < 0.001, ANOVA followed by Dunnett’s analysis).

Since either knockdown of CHC or treatment with dynasore causes a similar percentage of inhibition of VEGFR2 endocytosis (Fig. 1a,b), one would expect that these tools would exert a similar effect in VEGFR2 signalling. Yet, surprisingly, although knockdown of CHC had no effect in VEGF-induced activation of ERK1/2 (Fig. 2a), dynasore caused a robust inhibition at all time points of stimulation (Fig. 2b).

**Figure 3:**
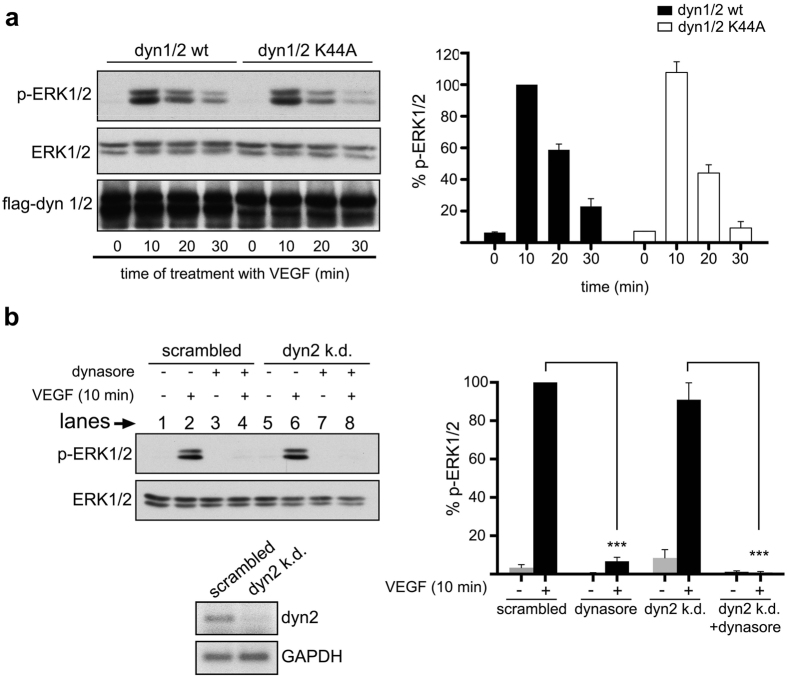
Dynasore-mediated inhibition of VEGF-to-ERK1/2 signalling is independent of dynamin. (**a**) HUVECs transduced with lentiviral vectors encoding dynamin (1 and 2) or dynamin K44A (1 and 2) were stimulated with VEGF for various time points, lysed and subjected to immunoblotting analysis using antibodies against the phosphorylated or total ERK1/2. Quantification of the effect of dynamin inhibition on the VEGF-induced phosphorylation of ERK1/2 is shown on the right of the immunoblots. The effect is considered non-significant (n = 3, mean ± S.D., ANOVA followed by Dunnett’s analysis). (**b**) Dynamin2 siRNAs treated HUVECs were serum starved for 2 h, incubated with 100 μM dynasore for 30 min and stimulated with VEGF for 10 min, lysed and processed for immunoblotting analysis using antibodies against the phosphorylated and total forms of ERK1/2. Immunoblots are representative of 3 independent experiments. Quantification is shown on the right of the immunoblots (n = 3, mean ± S.D., ***P < 0.001, ANOVA followed by Dunnett’s analysis). The efficiency of dynamin2 knockdown was assessed by semi-quantitative RT-PCR (see agarose gel at the bottom of **b**), as well as by immunofluorescence microscopy analysis of transferrin uptake (see S1b).

To elucidate this contradiction, at first we sought to test whether the effect of dynasore in VEGF signalling is mediated via inhibition of dynamin itself, or whether it is dynamin-independent. To this end, we tested the effect of overexpression of dynamin K44A, a dominant negative mutant of dynamin^38^, on ERK1/2 phosphorylation assays. Unlike dynasore, lentiviral expression of dynamin K44A had no substantial impact on VEGF-induced phosphorylation of ERK1/2 (Fig. 3a). Consistently with this finding, knockdown of dynamin-2, which is the ubiquitously expressed isoform of dynamin^39^, had also no effect on ERK1/2 phosphorylation (Fig. 3b, compare lanes 2 and 6) (the efficiency of dynamin K44A overexpression, or dynamin2 knockdown, to block transferrin uptake has been confirmed in Fig. S1a, or S1b, respectively). The above data (using dynamin K44A, or siRNAs against dynamin) are in complete consistency with the CHC knockdown experiment (Fig. 2a), suggesting that CME is dispensable for VEGF-to-ERK1/2 signalling. Importantly, the inhibitory effect of dynasore on ERK1/2 phosphorylation is observed even in dynamin-2 knockdown cells (Fig. 3b, compare lanes 2 and 4 with 6 and 8, respectively). Combined, these data indicate that the inhibitory effect of dynasore on VEGF-induced activation of ERK1/2 is independent of dynamin.

### Dynasore inhibits VEGF signalling in an endocytosis-independent manner

**Figure 4:**
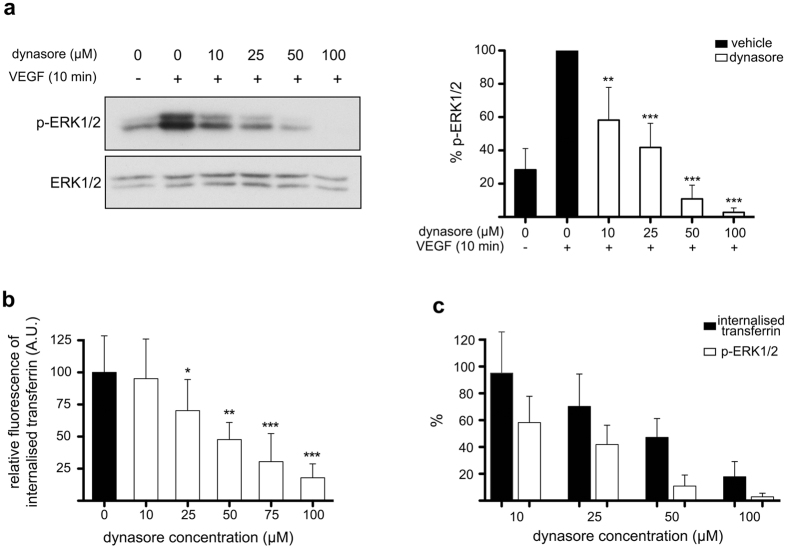
Dynasore inhibits signalling and endocytosis with different efficacies. (**a**) Immunoblotting analysis of the dose-dependent effect of dynasore on the VEGF-induced phosphorylation of ERK1/2 in HUVECs. Quantification of the phospho-ERK1/2 zones is shown on the right of the immunoblots (n = 3, mean ± S.D., **P < 0.05, ***P < 0.001, t-test). (**b**) Analysis of the dose-dependent effect of dynasore on CME. HUVECs were pre-treated (30 min) with different concentrations of dynasore and incubated with FITC-transferrin for 15 min, acid-washed, fixed and processed for immunofluorescence microscopy analysis (15 microscopy fields from 3 independent experiments were analysed, mean ± S.D., *P < 0.05, **P < 0.005, ***P < 0.001, t-test). (**c**) Dose response comparison of the inhibitory effect of dynasore on transferrin internalisation versus inhibition of VEGF-to-ERK1/2 phosphorylation. Graph bars express the % of internalised transferrin, or ERK1/2 phosphorylation, of dynasore treated cells relatively to vehicle treated cells.

To better comprehend the relationship between dynasore-mediated inhibition of endocytosis and its effect in signalling, we compared the dose-response of the drug in inhibiting VEGF signalling (Fig. 4a) versus inhibition of CME (Fig. 4b). Intriguingly, dynasore proved to be a more potent inhibitor of VEGF-induced activation of ERK1/2 than of CME of transferrin (Fig. 4c). These data suggest that the inhibitory effect of the drug in VEGF-induced activation of ERK1/2 is not due to a high dose effect, since it affects ERK1/2 phosphorylation at even lower concentrations than those required to inhibit CME. Furthermore, curiously, at 100 μM, which is the concentration needed to completely block endocytosis (Fig. 4b), dynasore inhibited ERK1/2 phosphorylation to levels that were even lower than the basal phosphorylation (i.e. in the absence of VEGF) of ERK1/2 (compare first with last lane of the blot in Fig. 4a), suggesting that dynasore inhibits also basal ERK1/2 activity, in a VEGF-independent manner. Collectively, the above observations (i.e., 1st, inconsistency between the effect of dynasore versus the effects of the knockdown or overexpression experiments in VEGF-to-ERK1/2 signalling, Figs 2a,b and 3a,b; 2nd, differential dose-response pattern for dynasore-mediated inhibition of transferrin uptake versus attenuation of VEGF-induced ERK1/2 phosphorylation, Fig. 4c; and 3rd, dynasore-mediated inhibition of the phosphorylation of ERK1/2 beyond the basal levels, Fig. 4a) indicate that dynasore impairs VEGF-to-ERK1/2 signalling in a manner that is independent of the inhibitory effect of the drug on VEGFR2 endocytosis, as well as independent of dynamin.

**Figure 5:**
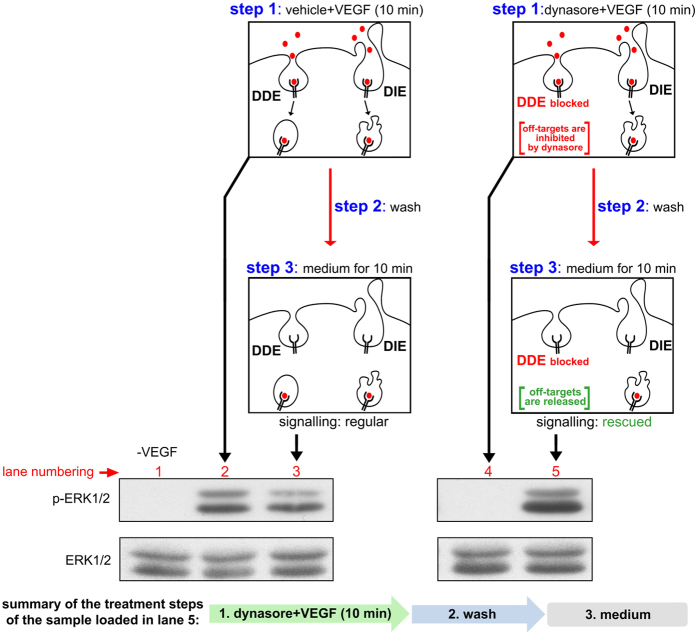
Dynasore-mediated inhibition of VEGF-to-ERK1/2 signalling is independent of DDE (“uncoupling experiment”). In step 1, HUVECs were treated with vehicle (left side) or dynasore (right side) and stimulated with VEGF (in the presence of vehicle or drug), for 10 min. The scheme (step 1, right side) illustrates that dynasore blocks both dynamin-dependent endocytosis (indicated in the scheme as “DDE blocked”) and the off-targets (indicated in the scheme as “off-targets are inhibited by dynasore”), while dynamin-independent endocytosis (DIE) remains unaffected. Then, the cells were either lysed and subjected to immunoblotting analysis, to test the effect of dynasore on ERK1/2 phosphorylation when the drug is continuously present (*see* lanes depicted by the long vertical arrow, lanes 2 and 4), or were acid washed (step 2), to remove membrane bound VEGF and dynasore (the effectiveness of acid wash treatment to remove VEGF from plasma membrane is shown in Fig. S4a), and processed to the next step. In step 3, cells were incubated in serum-free medium, for 10 min, to allow full recovery from dynasore, thereby releasing possible off-target effects of the drug (indicated in the scheme as “off-targets: released”, see step 3, right). As there is no ligand in this phase, DDE cannot resume despite removal of the drug (indicated in the scheme as “DDE blocked”). Thus, in lane 5, DDE does not take place throughout the whole experiment, even after removal of the drug (note that this is the main difference with the reversibility experiment shown in Fig. S2a, where VEGF is added after the removal of the drug, which rescues both DDE and the off-targets). Then, the cells were lysed and subjected to immunoblotting analysis using anti-phospho-ERK1/2 antibodies. Since DDE never took place in sample 5 (see above), recovery of the signal in this sample (compare lane 5 with lane 4), upon release of the off-targets, suggests that DDE is not essential for VEGF signalling; thus, the inhibitory effect of dynasore in lane 4 is due to the off-targets. At the bottom of the figure there is a schematic summary of the treatment steps of sample 5. Full-length blots are shown in Fig. S4b.

To further clarify whether the effect of dynasore is indeed endocytosis-independent, we developed a new protocol (which we named “uncoupling experiment”) that allowed us to assay VEGF-induced phosphorylation of ERK1/2 under conditions that the effect of the drug on endocytosis is uncoupled from its possible other effects, e.g. off-target effects^13^,^35^,^40^,^41^,^42^. As will become apparent below, key to the development of this protocol is the fact that the inhibitory effect of dynasore on VEGF-induced ERK1/2 phosphorylation is rapidly reversible, that is, after the drug is removed, VEGF is able to induce ERK1/2 phosphorylation (compare the last three lanes of the blot in Fig. S2a with the corresponding lanes 2–4 of the VEGF + vehicle treated cells) (the inhibitory effect of the drug on transferrin uptake is also reversible, Fig. S2b). The “uncoupling experiment” consists of three steps (drawn schematically in detail in Fig. 5). In step 1, HUVECs (pretreated with vehicle or dynasore for 30 min) are incubated for 10 min with VEGF alone (left side of Fig. 5), or with VEGF and dynasore (right side of Fig. 5) to prevent dynamin-dependent endocytosis (DDE). At this step (step 1), dynasore inhibits both DDE and the off-targets (illustrated in the scheme as “DDE blocked” and “off-targets are inhibited by dynasore”, respectively), while the receptor can still internalise through dynamin-independent endocytosis^18^ (noted in the scheme as “DIE”). In step 2, the cells are washed, to remove the drug. Subsequently, in step 3, the cells are incubated in plain medium for 10 min, to release any possible off-target effects of dynasore (lane 5). Note that, in this last step (step 3, lane 5), given that there is no ligand in the medium, DDE cannot resume despite removal of the drug (indicated in the scheme as “DDE blocked”), while the off-targets are now released (indicated in the scheme as “off-targets released”). Thus, in lane 5, DDE does not take place throughout the whole experiment (steps 1–3), even after removal of the drug. Interestingly, ERK1/2 phosphorylation was rescued completely in the sample of lane 5 (Fig. 5, compare lane 5 with lane 4; also, compare lane 5 with lane 3). Since DDE never took place in sample 5 (see above), recovery of the signal in this sample, upon release of the off-targets, suggests that DDE is not essential for VEGF signalling; thus, the inhibitory effect of dynasore in lane 4 is due to the off-targets. Notably, the data of the uncoupling experiment, suggesting that DDE is not important for activation of ERK1/2, are consistent with the experiments employing clathrin, or dynamin siRNAs or lentiviral overexpression of dynamin K44A (Figs 2a and 3a,b). Thus, dynasore inhibits VEGF-induced activation of ERK1/2 independently of its effects on endocytosis, as well as independently of dynamin (Fig. 3a,b).

### Dynasore impairs VEGF-induced calcium release

**Figure 6:**
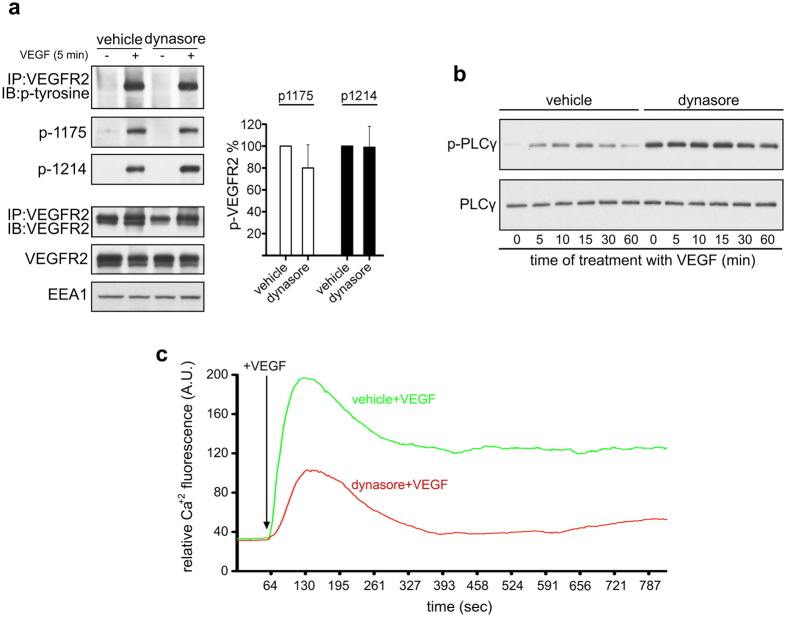
Dynasore inhibits VEGF-induced activation of calcium release. (**a**) Dynasore does not affect VEGF-induced phosphorylation of VEGFR2. Vehicle or dynasore treated serum deprived HUVECs were stimulated with VEGF for 5 min, lysed and subjected to immunoblotting analysis using antibodies against phosphorylated VEGFR2 at tyrosine 1175 or 1214. Total phosphorylation of the receptor was revealed by VEGFR2 immunoprecipitation and immunoblotting analysis of the precipitants using anti-phosphotyrosine antibodies. Quantification is shown on the right of the immunoblots (n = 3, mean ± S.D., t-test). (**b**) Dynasore potentiates phosphorylation of PLCγ. Vehicle or dynasore treated HUVECs were stimulated with VEGF (for the indicated time periods), lysed and analysed by immunoblotting using antibodies against phosphorylated and total PLCγ. (**c**) Time-course graph of VEGF-induced Ca^2+^ fluorescence in HUVECs treated with vehicle or dynasore in the presence of the Ca^2+^ indicator fluo-4 AM. The graph is representative of three independent experiments.

Since the inhibitory effect of dynasore on ERK1/2 activation (Figs 2b and 4a) is unrelated to both dynamin and VEGFR2 endocytosis (Figs 3, 4, 5), what is the mechanism by which dynasore inhibits ERK1/2 activation? In other words, what are the molecules that lie upstream of ERK1/2 that could be the “off-targets” of dynasore? Starting from the first molecule of the cascade, the receptor itself, we found that dynasore has no effect on total phosphorylation of VEGFR2, as well as on phosphorylation at position 1175 or 1214 (Fig. 6a). Subsequently, we tested whether PLCγ, which lies downstream of VEGFR2^14^, is the target of dynasore. At first, we confirmed that PLCγ is indeed absolutely essential for VEGF-induced activation of ERK1/2 (Fig. S3). Then, curiously, we found that dynasore augments the basal phosphorylation levels of PLCγ (Fig. 6b), even beyond the levels induced by VEGF (Fig. 6b). However, the subsequent event downstream of PLCγ phosphorylation, i.e. increase of cytoplasmic calcium levels^14^, was substantially attenuated by treatment with dynasore (Fig. 6c), despite the over-phosphorylation of PLCγ (Fig. 6b) (see discussion for possible explanation of this finding). Collectively, the above data imply that dynasore mediated inhibition of VEGF-induced activation of ERK1/2 could be due, at least in part, to an interference of the drug with the release of calcium (Fig. 6c), while it is independent of clathrin- or dynamin-dependent endocytosis of VEGFR2 (Figs 2a, 3, 4 and 5).

## Discussion

Given the importance of endocytosis in regulating receptor signalling^1^,^2^,^3^,^4^, the role of VEGFR2 internalisation has been a subject of intense study^18^,^19^,^21^,^22^,^23^,^24^,^26^,^27^,^28^,^29^,^31^,^37^,^43^. In the absence of VEGF, the main internalisation route of VEGFR2 is clathrin-mediated^20^,^37^. The role of this constitutive route is to protect the receptor against plasma membrane shedding^37^. On the other hand, in the presence of VEGF the preferred route of entry of the receptor is macropinocytosis^18^, which plays important role in VEGF-mediated signalling and endothelial functions^18^. Despite the preference for macropinocytosis^18^ (in the presence of VEGF), part of the receptor is also internalised via CME^18^,^19^,^20^,^21^,^22^,^23^,^24^,^25^,^26^,^27^,^28^,^29^,^30^,^31^,^32^. Yet, it is still debated whether CME of VEGFR2 plays a positive or a negative role in VEGF signalling. Thus, on one hand, knockdown of clathrin, or dynamin, or epsins had either no effect or augmented VEGF-induced activation of ERK1/2 or Akt^18^,^21^,^22^,^24^,^28^,^29^, suggesting that CME is not essential for VEGF-mediated activation of ERK1/2; yet, in contrast to the above data, other studies have pointed out that dynasore-mediated inhibition of CME inhibits VEGF signalling^23^,^26^,^33^,^34^. In the present study, several lines of evidence suggest that although dynasore is specific in inhibiting CME of VEGFR2 (Fig. 1b), dynasore-mediated inhibition of VEGF-induced activation of ERK1/2 (Figs 2b and4a) is due to an off-target effect and not the result of interference with endocytosis. First, there is a differential dose-response pattern of dynasore-mediated inhibition between transferrin uptake and VEGF-induced signalling (Fig. 4c). Second, using a newly developed “uncoupling experiment”, we found that the effect of dynasore on VEGF-induced activation of ERK1/2 is independent of VEGFR2 endocytosis (Fig. 5). Third, unlike dynasore, knockdown of clathrin or dynamin, or overexpression of dynamin K44A had no effect on VEGF-induced activation of ERK1/2 (Figs 2aand 3a,b), which is in accordance with previous studies^18^,^21^,^22^,^24^,^29^. Finally, fourth, the effect of dynasore in VEGF-to-ERK1/2 signalling persisted even in dynamin-knockdown cells (Fig. 3b), suggesting that the inhibitory effect of dynasore on VEGF-induced activation of ERK1/2 is dynamin-independent. Notably, a close structural analogue of dynasore, dyngo4a^44^, exerted a similar inhibitory effect on VEGF-induced activation of ERK1/2 (Fig. S5).

In endothelial cells, dynasore inhibits ERK1/2 activation downstream of several growth factors, such as VEGF, HGF, and FGF2^26^. Thus, it appears that the effect of dynasore on ERK1/2 activation is not ligand-specific. These observations, together with our finding that the effect of dynasore on VEGF-induced phosphorylation of ERK1/2 is unrelated to receptor endocytosis, suggest that the off-target of dynasore is a common signalling molecule that lies in between the above growth factors and ERK1/2. Our finding that dynasore inhibits VEGF-induced calcium release, a signalling event that lies upstream of ERK1/2^14^, implies that the effect of the drug on calcium levels could be responsible, at least in part, for the impact of the drug on VEGF-to-ERK1/2 signalling.

Given the inhibitory effect of dynasore on cytoplasmic calcium levels (Fig. 6c), it was curious to note that dynasore induces the phosphorylation of PLCγ in HUVECs (Fig. 6b), as well as in other cell types (Fig. S6a) (note that dynamin-mediated over-phosphorylation of PLCγ was dynamin-independent, as it occurred also in cells treated with siRNAs against dynamin, Fig. S6b). To explain these data, i.e. inhibition of calcium release in spite of the over-phosphorylation of PLCγ, one could hypothesise that, (1), dynasore may inhibit the catalytic phospholipase activity of (an otherwise hyper-phosphorylated) PLCγ, or, (2), it may limit the hyper-phosphorylated PLCγ in a subcellular area that is poor in PIP2, thus preventing PLCγ to transmit downstream signalling, or, (3), it may even interfere with the release of calcium from the intracellular stores.

Besides interfering with calcium release, dynasore may have additional off-target(s) in the VEGF-ERK1/2 cascade, which may be the cause for the severe effect on ERK1/2 phosphorylation (Figs 2b and4a). These off-target(s) might be molecule(s) lying downstream of calcium release (e.g. PKC, Raf, MEK, ERK1/2), or/and molecule(s) in the Ras-ERK1/2 axis (e.g. GRB2, SOS, Ras). In fact, dynasore inhibits the basal phosphorylation levels of Raf 26. Regardless of the exact mechanism, our data suggest that dynasore inhibits VEGF-induced ERK1/2 activation (Figs 2b and 4a) in a manner that is unrelated to receptor endocytosis (shown by clathrin knockdown, Fig. 2a; dynamin K44A overexpression, Fig. 3a; dynamin knockdown, Fig. 3b; and by the uncoupling experiment, Fig. 5), as well as independent of dynamin (Fig. 3a,b).

Inhibitors of dynamin, as well as of other trafficking regulators, are powerful tools to efficiently block endocytosis^13^,^35^, especially in the case of difficult to transfect cells^45^. These tools offer the advantage of a rapid effect, thereby avoiding the manifestation of compensatory trafficking pathways^46^. However, the fact that they are small molecules that could bind to multiple targets, raises the concern that they may exert off-target effects^13^,^35^. The data shown here suggest that dynasore exerts an off-target effect on the PLCγ-ERK1/2 cascade. Thus, previous findings that relied solely on dynasore, as a tool to investigate the role of dynamin-dependent endocytosis in signalling, should be revisited. The novel assay (“uncoupling experiment”) that we describe in the present study can now serve as a universal tool for assessing whether the effect of a drug in signalling (or in other cellular functions) is strictly due to interference with endocytosis or due to an off-target effect of the drug.

## Methods

### Reagents and antibodies

Recombinant human VEGFΑ_165_ was obtained from Immunotools. Mouse anti-VEGFR2 extracellular domain monoclonal antibodies were from Abcam, whereas the rabbit monoclonal and polyclonal antibodies were from Cell Signalling and Santa Cruz Biotechnology, respectively. Fluorescein isothiocyanate-conjugated transferrin was from Invitrogen. The anti-actin antibody was from Millipore, whereas the anti-clathrin heavy chain antibody was from BD Biosciences. The rabbit polyclonal antibody against Early Endosome Antigen 1 (EEA1) was kindly provided by Marino Zerial (MPI-CBG, Dresden, Germany). The antibodies against ERK1/2, p-ERK1/2, PLCγ, p-PLCγ and p-VEGFR2 (1175) were from Cell Signalling whereas the antibody against p-VEGFR2 (1214) was from Invitrogen. Secondary antibodies coupled to Alexa fluorophores were from Invitrogen, while the HRP conjugated antibodies were from Jackson Immunoresearch. All other reagents were obtained from Sigma-Aldrich, unless stated otherwise.

### Cell culture and transfection or infection

Human Umbilical Vein Endothelial Cells (HUVECs) were isolated, cultured and transfected as described previously^47^. HeLa cells were cultured in DMEM medium supplemented with 10% FCS and 1% glutamine whereas BE(2)-C cells were cultured in DMEM/F-12 medium supplemented with 10% FCS. The siRNAs for human clathrin heavy chain (5′GGGUGCCAGAUUAUCAAUUtt3′) and PLCγ (5′CCCUUACCACCAAGAUCAAtt3′ and 5′GGGAAACAAAGUUUACAUUtt3′) were from Ambion, whereas the siRNAs for human dynamin-2 (5′CAUGCCGAGUUUUUGCACUtt3′) and control siRNA (Random DS) were from Biospring. All knockdown experiments were carried out using 50 nmol/L of siRNAs. For knock down of PLCγ, a mixture of 2 siRNΑs (25 nmol/L each) was used. Transduction of HUVECs with lentiviral vectors and semi-quantitative RT-PCR was performed as described previously^18^.

### Indirect immunofluorescence microscopy

HUVECs were cultured in 35-mm diameter plastic dishes (appropriate for microscopy, by Ibidi), coated with collagen type I. To monitor the internalisation fate of cell surface pool of VEGFR2, 2 h serum-starved HUVECs were transferred to 4 °C for 30 min and incubated for 1 h with 10 μg/ml of mouse anti-VEGFR2 extracellular domain antibodies. Cells were treated for 30 min with vehicle (DMSO) or dynasore (100 μM) and transferred to 37 °C for 15 min in the presence of 50 μg/ml fluorescein isothiocyanate-conjugated transferrin and VEGF (50 ng/ml). Cells were acid-washed twice (ice cold M199 medium, pH 2.0), fixed and processed for immunofluorescence microscopy. The above protocol was also applied to siRNAs treated cells.

Indirect immunofluorescence and analysis by confocal microscopy was employed as previously described^48^. Images were captured using a Leica TCS SP5 II scanning confocal microscope and a Leica 63X HCX PL APO 1.4 NA objective. Data were subsequently processed in LAS AF according to the manufacturer guidelines.

### Immunoprecipitation

To access the tyrosine phosphorylation status of VEGFR2, serum deprived cells (4 h) were treated for 30 min with vehicle (DMSO) or dynasore (100 μM), in M199 medium, and stimulated with 50 ng/ml VEGF for 5 min. Cells were transferred to 4 °C, washed 3X with ice cold Ca^2+^/Mg^2+^ HBSS buffer (supplemented with 300 μΜ vanadate, 600 μmol/L Η_2_O_2_ and 1 mmol/L phenylmethanesulfonyl fluoride) and scraped in ice cold lysis buffer (50 mmol/L Tris, pH 7.4, 150 mmol/L NaCl, 1% Triton X-100, 1% NP-40, 1 mmol/L phenylmethylsulfonyl fluoride, 300 μmol/L vanadate, 600 μmol/L Η_2_O_2_ and protease inhibitors). Extracts were centrifuged at 16.000 × g for 30 min, supernatants were pre-cleared with protein A-agarose beads and were incubated with 10 μg of rabbit polyclonal anti-VEGFR2 antibodies, overnight, at 4 °C, under rotation. Immuno-complexes were precipitated using protein A-agarose beads and equal volumes of eluted proteins were subjected to western blotting analysis using a mouse anti-phosphotyrosine antibody. To normalize the levels of precipitated receptor between different samples, equal volumes of precipitates were analysed by western blotting using a rabbit monoclonal anti-VEGFR2 antibody.

### Reversibility of dynasore inhibition

To test the reversibility of the inhibition of dynasore in VEGFR2 signalling, the following experimental procedure was applied. Serum deprived cells (2 h) were treated with 100 μM dynasore for 30 min. At the end of the treatment, cells were washed 3x with Ca^2+^/Mg^2+^ HBSS and the medium was replaced with serum-free M199 medium. 10 min following medium replacement, cells were stimulated with VEGF (50 ng/ml, 10 min time intervals), lysed and analysed by immunoblotting using antibodies against ERK1/2 and phosphorylated ERK1/2. Vehicle treated cells were processed and analysed as above. Dynasore treated cells, but not stimulated with VEGF, were lysed (10 min intervals upon dynasore withdrawal) and analysed in parallel. To verify the functionality of the drug under the above experimental conditions, dynasore treated cells were stimulated for 10 min with VEGF, in the presence of dynasore, lysed and analysed by western blotting.

### Uncoupling experiment

The uncoupling experiment, which uncouples the effect of a drug in trafficking from possible off-target effects of the drug, consists of three steps (see scheme in Fig. 5). In step 1, serum starved HUVECs (2 h) were treated with vehicle or dynasore (100 μM) for 30 min, followed by stimulation with VEGF (50 ng/ml) for 10 min. Then, the cells were either lysed and analysed by western blotting, or processed in step 2, where they were acid-washed twice (M199 medium, pH 2.0) followed by 3 washes with Ca^2+^/Mg^2+^ HBSS. Then, in step 3, the cells were incubated in fresh serum-free medium for 10 or 20 min (to release any off-target effects of the drug), lysed and analysed by western blotting using antibodies against ERK1/2 or phosphorylated ERK1/2. Although during the 10 and 20 minute incubation time the receptor may have moved to later endosomal compartments, the conclusions regarding the importance of the different internalisation routes in signalling are not affected.

### Intracellular Ca^2+^ measurements

Serum starved HUVECs were loaded for 30 min at 37 °C with the Ca^2+^ indicator dye Fluo-4-AM (Invitrogen) in serum-free medium, according to manufacturer instructions, in the presence of vehicle or 100 μΜ dynasore. Cells were rinsed 3x with HBSS and the medium was replaced with pre-warmed microscopy solution. Cells were transferred to a 37 °C chamber of the confocal microscope and basal Ca^2+^ fluorescence was monitored for 3 min, upon excitation of cells at 480 nm. Subsequently, the cells were stimulated with 50 ng/ml VEGF and the levels of intracellular Ca^2+^ were recorded for 15 min (1.3 sec time lapses). Correction of background fluorescence and analysis of relative fluorescence intensity of confocal images was performed in ImageJ.

### Quantifications and statistical analysis

The quantification of immunoblots and immunofluorescence images was performed using the ImageJ software. Statistical differences were evaluated using the student t-test, for two-group comparison, or analysis of variance (ANOVA) followed by Dunnett’s post hoc analysis for comparisons of more than two groups.

## Additional Information

**How to cite this article:** Basagiannis, D. *et al*. Dynasore impairs VEGFR2 signaling in an endocytosis-independent manner. *Sci. Rep.* **7**, 45035; doi: 10.1038/srep45035 (2017).

**Publisher's note:** Springer Nature remains neutral with regard to jurisdictional claims in published maps and institutional affiliations.

## Supplementary Material

Supplementary Figures
