# Supplementary material for: Dynasore impairs VEGFR2 signalling in an endocytosis-independent manner
Source: Sci Rep. 2017 Mar 22;7:45035. doi: 10.1038/srep45035 (PMC5361198; doi:10.1038/srep45035)
Supplement: Supplementary Figures [file srep45035-s1.pdf]

Dynasore impairs VEGFR2 signalling in an endocytosis-independent manner

Dimitris Basagiannis[1,2], Sofia Zografou[1], Katerina Galanopoulou[1,2] and Savvas Christoforidis[1,2,*]

Figure S1

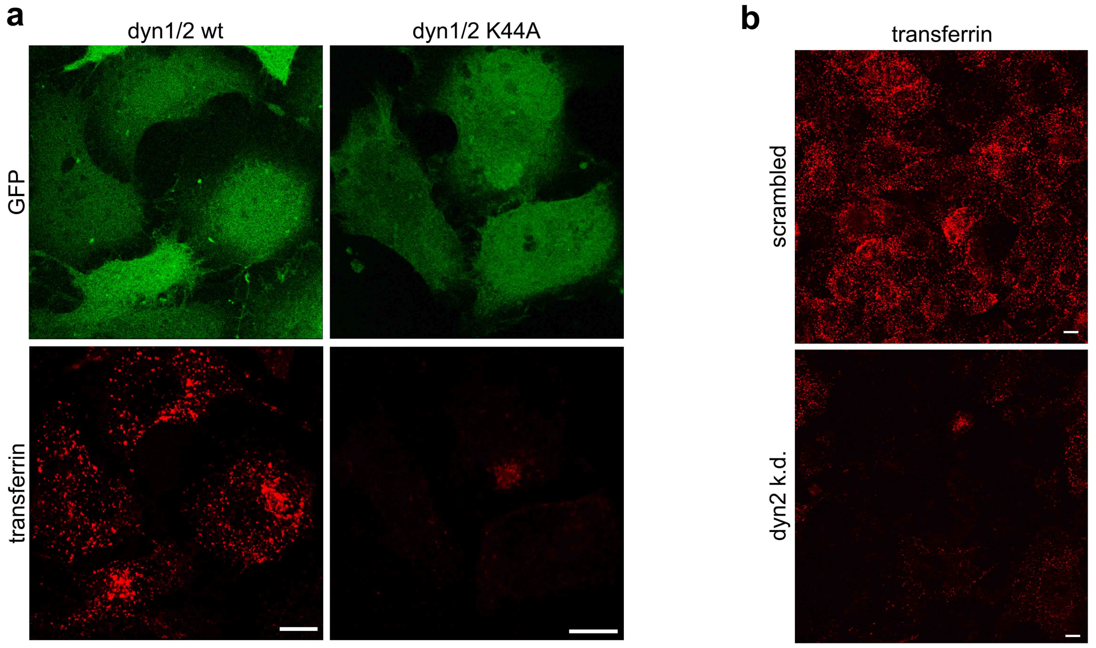

Figure S1. Overexpression of dynamin K44A, or knockdown of dynamin 2, blocks CME.

(**a**) HUVECs transduced with lentiviral vectors encoding dynamin wt (1 and 2) or dynamin K44A (1 and 2) were incubated with 50 ng/ml TRITC-transferrin for 15 min, acid washed to remove membrane bound transferrin, fixed and subjected to immunofluorescence microscopy analysis. Scale bars represent 10 μm. GFP expression was used to assess the efficiency of lentiviral transduction (see methods). Immunofluorescence microscopy images are representative of 3 independent experiments. (**b**) HUVECs treated with siRNAs against dynamin2 were incubated with 50 ng/ml TRITC-transferrin for 15 min, acid washed to remove membrane bound transferrin, fixed and subjected to immunofluorescence microscopy analysis. Scale bars represent 10 μm. Immunofluorescence microscopy images are representative of 3 independent experiments.

Figure S2

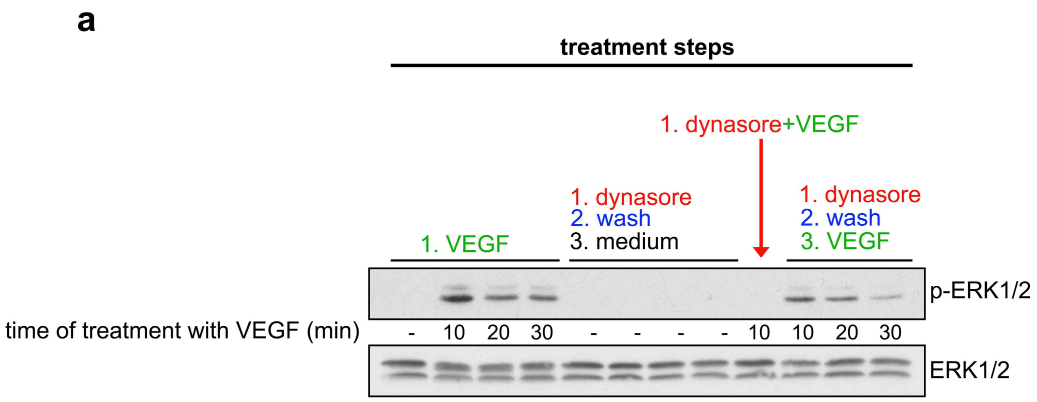

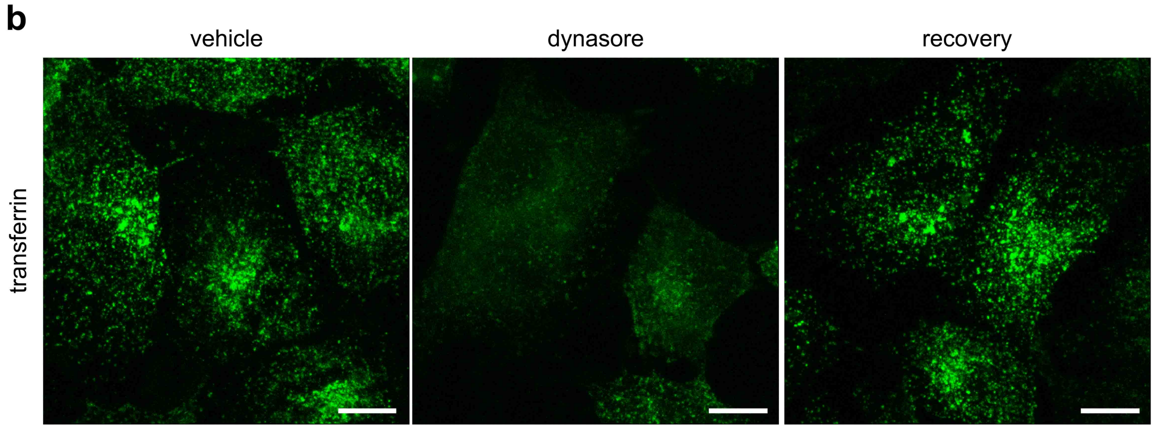

Figure S2. The inhibitory effects of dynasore on ERK phosphorylation and on CME are reversible.

(**a**) Dynasore-mediated inhibition of ERK1/2 phosphorylation is reversible. In the last 3 lanes of the immunoblot, serum deprived HUVECs were treated with dynasore for 30 min, washed, incubated in serum free medium for 10 min, stimulated with VEGF for the indicated time intervals, and subjected to immunoblotting analysis using antibodies against phosphorylated ERK1/2. To test that the reversibility of the effect of dynasore still depends on activation by VEGF, identically treated samples (as above), but without VEGF, were analysed in parallel (middle 4 lanes). Control samples of vehicle treated, VEGF stimulated cells, were analysed in parallel (first 4 lanes). The arrow indicates a sample that was stimulated in the presence of dynasore, without subsequent washes (to control the effectiveness of the drug). Equal loading was tested with total protein levels of ERK1/2.

(**b**) Dynasore-mediated inhibition of CME is reversible. To access the efficiency of CME inhibition by dynasore, HUVECs were treated with vehicle (*left image*) or 100 μM dynasore (*middle image*) for 30 min, incubated with 50 ng/ml FITC-transferrin for 15 min, acid washed to remove membrane bound transferrin, fixed and subjected to immunofluorescence

microscopy analysis. To test the reversibility of dynasore-mediated blockage of CME (*right image*), HUVECs were treated with 100μM dynasore for 30 min, washed and incubated in serum free medium for 10 min. Subsequently, cells were incubated with 50 ng/ml FITC-transferrin for 15 min, acid washed to remove membrane bound transferrin, fixed and subjected to immunofluorescence microscopy analysis. Scale bars represent 10 μm. Immunofluorescence microscopy images are representative of 2 independent experiments.

Figure S3

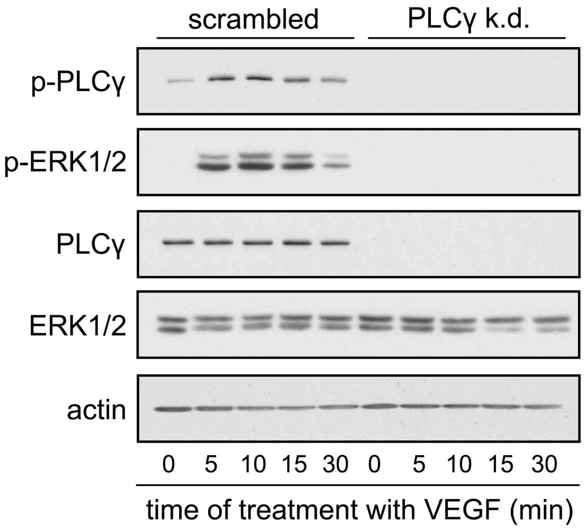

Figure S3. VEGF-induced activation of ERK1/2 depends on PLCγ.
HUVECs were transfected with siRNAs against PLCγ, were serum-starved for 2h and stimulated with VEGF for the indicated time points. Cells were lysed and analysed by immunoblotting using antibodies against VEGFR2, actin and total or phosphorylated forms of ERK1/2 or PLCγ.

Figure S4

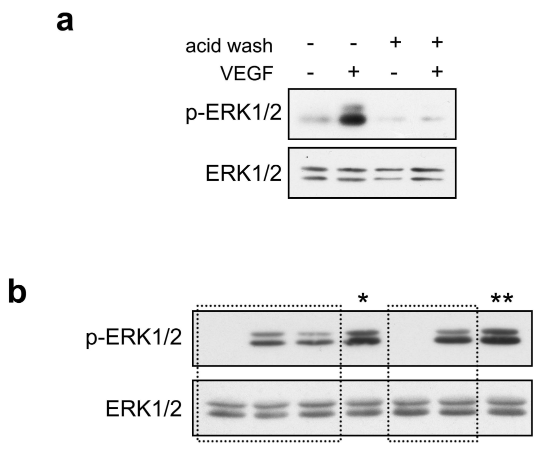

Figure S4. (**a**) Test of the effectiveness of acid wash treatment to remove VEGF from plasma membrane VEGFR2. Serum deprived HUVECs were transferred to 4^0C and incubated for 10 min with ice cold serum-free M199 medium supplemented with or without 50 ng/ml VEGF, to allow binding of VEGF to cell surface receptors. Subsequently, cells were washed twice with ice-cold M199 medium (control cells, lanes 1 and 2 in the blots) or with ice-cold M199 medium pH 2.0 (acid wash, lanes 3 and 4 in the blots), to strip VEGFR2-bound VEGF, followed by 3 washes with Ca^{2+}/Mg^{2+} HBSS. Then, the cells were transferred to 37^0C for 10 min (to allow activation of ERK1/2, in response to the remaining VEGF at the plasma membrane), lysed and processed for immunoblotting analysis using antibodies against the phosphorylated and total forms of ERK1/2. Compared to the strong phosphorylation of ERK1/2 in control cells (lane 2), acid wash treatment abolishes VEGF-induced phosphorylation of ERK1/2 (lane 4), which verifies that treatment of the cells with low-pH medium efficiently strips VEGF from the cell surface. Immunoblots are representative of 3 independent experiments.

(**b**) Full-length blots of Figure 5. Dotted boxes show the parts of the blots that are presented in Fig. 5. The lanes indicated by one or two asterisks, which have been omitted in the blots of Figure 5, correspond to samples treated for 20 min with medium containing vehicle+VEGF, or dynasore+VEGF, respectively, at step 3.

Figure S5

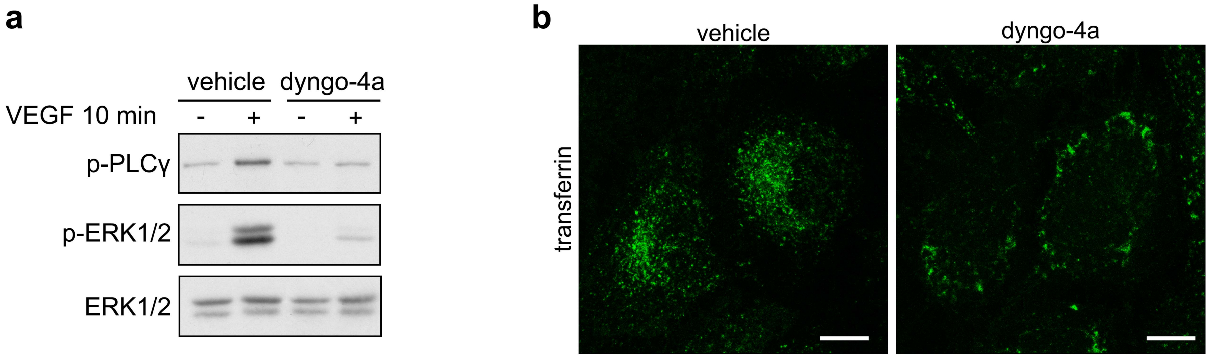

Figure S5. Treatment of endothelial cells with dyngo-4a attenuates VEGF signaling. (**a**) HUVECs were serum starved for 2h and were treated with vehicle (DMSO) or dyngo-4a (30 μM) for 30 min. Subsequently, the cells were stimulated with VEGF (50 ng/ml) for 5 min, lysed and subjected to immunoblotting analysis using antibodies against the phosphorylated forms of PLCγ or ERK1/2. Equal protein loading was tested by immunoblotting against total ERK1/2. Immunoblots are representative of 3 independent experiments. (**b**) Dyngo-4a inhibits CME. HUVECs treated with vehicle or dyngo4a (30 μM) for 30 min were incubated with 50 ng/ml TRITC-transferrin for 15 min, acid washed (to remove membrane bound transferrin), fixed and subjected to analysis by laser confocal microscopy. Scale bars represent 10 μm. Microscopy images are representative of 3 independent experiments.

Figure S6

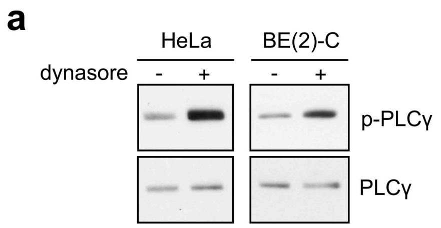

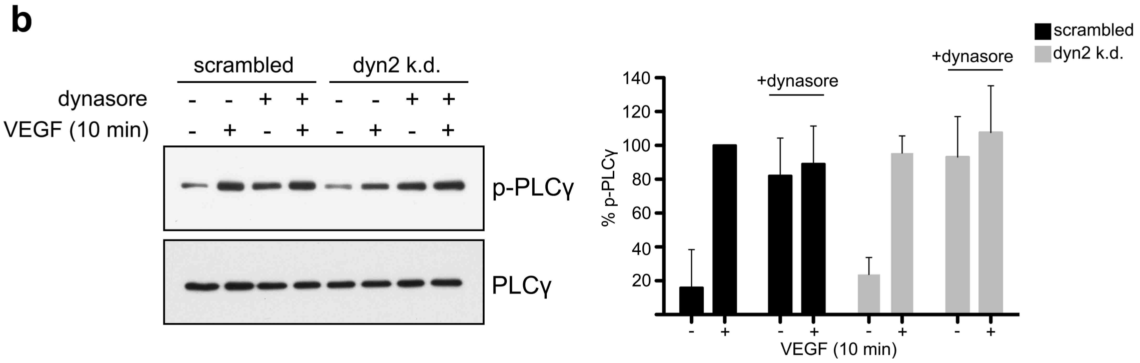

Figure S6. Dynasore potentiates the basal phosphorylation levels of PLCγ in different cell types, in a dynamin-independent manner.

(**a**) Dynasore potentiates the basal phosphorylation levels of PLCγ in different cell types. HeLa or BE(2)-C cells were serum starved for 4h and treated with 100 μM dynasore for 30 min. Subsequently, cells were lysed and processed for western blotting analysis using antibodies against the phosphorylated and total form of PLCγ. Immunoblots are representative of 3 independent experiments. (**b**) Dynasore potentiates the basal phosphorylation levels of PLCγ in a dynamin-independent manner. Dynamin2 siRNAs treated HUVECs were serum starved for 2h, incubated with 100 μM dynasore for 30 min and stimulated with VEGF for 10 min, lysed and processed for immunoblotting analysis using antibodies against the phosphorylated and total forms of PLCγ. Quantification is shown on the right of the immunoblots (n=3, mean±S.D, t-test). Note that the samples of the immunoblots shown in panel **b** originate from the same experiment, and were run in the same immunoblot, as the samples of Fig. 3b. The efficiency of dynamin2 knockdown was assessed by semi-quantitative RT-PCR (see Fig. 3b, bottom panel) and by immunofluorescence microscopy analysis of transferrin uptake (see Fig. S1b).
